# Copper Analysis by Two Different Procedures of Sequential Extraction after Electrodialytic Remediation of Mine Tailings

**DOI:** 10.3390/ijerph16203957

**Published:** 2019-10-17

**Authors:** Andrea Lazo, Pamela Lazo, Alejandra Urtubia, María Gabriela Lobos, Claudia Gutiérrez, Henrik K. Hansen

**Affiliations:** 1 Departamento de Ingeniería Química y Ambiental, Universidad Técnica Federico Santa María. Avenida España 1680, Valparaíso 2390123, Chile; andrea.lazo@usm.cl (A.L.); alejandra.urtubia@usm.cl (A.U.); claudia.gutierrez@usm.cl (C.G.); henrik.hansen@usm.cl (H.K.H.); 2 Instituto de Química y Bioquímica, Facultad de Ciencias, Universidad de Valparaíso, Avenida Gran Bretaña 1111, Playa Ancha, Valparaíso 2360102, Chile; gabriela.lobos@uv.cl; 3 Centro Científico Tecnológico de Valparaíso, CCTVaL, Avenida España 1680, Valparaíso 2390123, Chile; 4 Centro de Biotecnología Daniel Alkalay, CBDAL, General Bari 699, Valparaíso 2390123, Chile

**Keywords:** leaching, sequential extraction, copper, fractions, electrodialytic remediation

## Abstract

The analysis of Cu distribution in pre-treated mine tailings after electrodialytic remediation was carried out by using two methods of sequential extraction. The initial content of copper in the tailings was 1109 mg Cu/kg of dry tailing, where close to 40% of the sample in weight corresponded to a soluble fraction. The tailing was treated with a leaching solution for 24 h. Three different solutions were tested: H_2_SO_4_ + HNO_3_ with pH = 1.9; H_2_SO_4_ + HNO_3_ with pH = 4.2; and NH_4_Cl 0.8 mol/L with pH = 5.5. After that, electrodialytic remediation experiments were carried out using an electric field of 2.7 V/cm for 15 days. The best performance for the complete cell was obtained with H_2_SO_4_ + HNO_3_ solutions, with a copper removal efficiency in the range of 62% to 67% and a current efficiency between 6% and 9%. The results of the remaining copper concentration between anode and cathode, from both procedures of sequential extraction, showed similar trends. The differences were mainly attributed to the use of different extractant solutions and extraction times. Soluble and exchangeable fractions were easily removed, with efficiencies higher than 80%. The lowest copper removal efficiency was obtained with NH_4_Cl 0.8 mol/L.

## 1. Introduction

Mining is one of the most important economic activities in Chile, however, it generates large amounts of waste products, particularly when processing sulfide ores from porphyry copper deposits [1]. The main residue from mining activities are tailings, which consist of a mixture between fine-grained ground-up rock and processed water with dissolved metals and reagents that remain after the minerals of economic importance have been extracted. Their disposal is one of the major important environmental issues in a copper mine [2,3].

Several studies have been carried out about the application and usefulness of electrodialytic treatment to remove toxic metals such as Cu, As and Pb from mine tailings. Electrodialytic remediation (EDR) is a combination between the electro-kinetic method and electrodialysis in which ion exchange membranes are used to separate the soil, or other contaminated matrices, and the solutions where the metals are concentrated [4]. Generally, EDR experiments are carried out at a constant electric field or constant current [5,6,7,8].

To achieve an increase in the solubility and mobility of copper ions, highly acidic conditions are necessary. For that reason, to facilitate the electrodialytic process, leaching solutions are used [9]. Some solutions employed by the industry are a mixture between hydrochloric acid and nitric acid adjusted to different pH. Also, ammonium chloride, which allows the forming of chemical complexes with chloride and copper, is used. In both cases, the removal of copper would favor the copper dissolution [8,10,11].

It is known that the toxicity and behavior of the elements depend on their chemical form in nature. Sequential extraction procedures are considered appropriate to clear up the fractionation of toxic metals between different forms and have been successfully applied to tailing analysis [9]. This kind of procedure is still used, despite its drawbacks, because they provide information on the fractionation of metals in the different lattices of the solid sample, which serves as a good compromise as it gives information on environmental contamination risks [12].

Several studies about the potential environmental risk of metallic elements have been carried out in other countries with copper mining activities. In Morocco, Yassir et al., 2015, studied the different associations of Cd, Cu, Pb and Zn in the mine tailings of the Draa Lasfar mine. The results suggested that Cd and Cu were mainly associated with a more mobile fraction, Pb with a less mobile fraction and Zn was essentially bound to Fe-Mn oxides [13]. The study of Pourret et al., 2016, was focused in the analysis of the fractionation and mobility of Co and Cu in contrasting soils (soils, mine tailings, smelting waste disposal) from the Katanga mine [14]. In the research conducted by Chen et al., 2018, the acid and neutralization potential and the possibility for producing acid mine drainage from tailings collected from a reservoir at the Huogeqi mine, located in Mongolia, China, was evaluated. The results showed an insignificant quantity of acid mine drainage, where Cu in the tailing sample existed in an oxidizable form [15].

Usually the sequential extraction methods divide the element into an exchangeable fraction, carbonate-bound fraction, Fe-Mn oxide-bound fraction, organic and sulfide fraction and residual fraction [16,17,18,19,20]. Some modifications have been proposed, such as the application of a sequential extraction procedure, which divides the sample into six fractions: water, soluble, exchangeable, carbonate-bound, Fe-Mn oxide-bound, organic-bound and residual fractions for the analysis of Cu, Cd, Ni and Zn in contaminated soils [21]. Other studies used a seven-fraction sequential extraction procedure to analyze the different associations of Cu in mine tailings. This procedure allows distinguishing between: water-soluble fractions, exchangeable fractions, oxy-hydroxides and oxides of Fe(III) in different steps, organic and secondary Cu-sulfide fractions, primary sulfide and residual silicate fractions. The optimization of the method has been developed for the analysis of arsenic, copper and vanadium, among others [21,22,23].

The comparison between two sequential extraction procedures has been carried out by Anju et al. [9], who applied a modified procedure proposed by the Community Bureau of Reference (BCR) and the Tessier’s scheme to the fractionation of Cd, Zn and Pb in mine tailings. In addition, Dold [21] applied two types of sequential extraction procedures adapted according to the mineralogy of the tailings for copper fractionation.

The aim of this work is to determine the amount of removed copper from mine tailings by electrodialytic remediation and the effect of applying different leaching solutions before the electrodialytic treatment. In addition, this research studies the copper association with different fractions in the mine tailings through the cell, before and after the leaching and electrodialytic treatment. The determination of copper associated with each fraction is carried out by means of two different procedures of sequential extraction and the congruence of the results from both methods is analyzed.

## 2. Materials and Methods

### 2.1. Electrodialytic Remediation

Tailing samples from the El Teniente copper mine located in the VI Region of Chile were used in this study. The tailings were mixed with a pre-treatment solution in a proper ratio to obtain a humidity of 20%, stepping for 24 h before the electrodialytic experiments, with the aim to enhance the mobility of certain ions. Three different pre-treatment solutions were used: (1) H_2_SO_4_ + HNO_3_ (2:1 vol.) pH = 1.9; (2) H_2_SO_4_ + HNO_3_ (2:1 vol.) pH = 4.2; and (3) NH_4_Cl 0.8 mol/L with pH = 5.5.

The electrodialytic experiments were carried out for 360 h with a total voltage difference between the anode and cathode of 20 V. The data were acquired online with a digital multimeter model UT60A, UNI-T (Uni-Trend Technology Co., Ltd., Shenzhen, China). For all experiments, a custom-made acrylic cylindrical cell [8,24] with a sample compartment 8 cm in length and 5 cm in inner diameter was used. The volumes of the anolyte and catholyte compartments were 144 mL each. Cation exchange membrane CMI-7000 and anion exchange membrane AMI-7001 from Membranes International Inc. (Ringwood, NJ, USA) were used. The anode and cathode were made of titanium. The catholyte and anolyte solutions corresponding to sulfuric acid solutions 0.25 mol/L and 0.025 mol/L, respectively, were recirculated by peristaltic pumps Model MD-6-230GSO from Iwaki (Boston, MA, USA). In order to keep the pH value constant, sulfuric acid was added dropwise to the catholyte.

All reagents were analytical grade, Merck, Kenilworth, NJ, USA: H_2_SO_4_ 95%–97% (ISO grade); HNO_3_ 65% (Ph Eur, ISO); and NH_4_Cl and HCl 37% (ACS, Ph Eur, ISO).

#### 2.1.1. Copper Removal Efficiency

The effectiveness of electrodialytic treatment for copper removal can be quantified through copper removal efficiency calculation using Equation (1):

(1) $$\eta_{Cu\;removal,i}\left(\%\right)=\left(\frac{m_{Cu\;initial,i}-m_{Cu\;final,i}}{m_{Cu\;initial,i}}\right)\cdot 100$$

where $\eta_{Cu\;removal,i}$ is the removal efficiency for copper associated to fraction i, $m_{Cu\;initial,i}$ is the initial concentration of copper associated to fraction i and $m_{Cu\;final,i}$ is the final concentration of copper associated to fraction i. The copper mass was expressed in g.

The removal copper efficiency of total cells was calculated with the mass of copper present in the tailing at the beginning and at the end of each experiment in the complete cell.

#### 2.1.2. Copper Efficiency

Current efficiency is defined as the ratio between the mass of copper effectively transported and the theoretical mass that should be transported. Current efficiency was calculated using Equation (2):

(2) $$\eta_{current}\left(\%\right)=\left(\frac{M_{Cu\;exp}}{M_{Cu\;theo}}\right)\cdot 100$$

where $\eta_{current}$ is the current efficiency, $M_{Cu\;exp}$ is the mass of copper transported in each experiment and $M_{Cu\;theo}$ is the theorical mass of copper predicted by Faraday Law at 100% efficiency. The copper mass was expressed in g.

### 2.2. Sequential Extraction Procedures

The analysis of treated tailings was carried out by two different procedures of sequential extraction. The first procedure, corresponding to Tessier [19,20], divides metals into six fractions, i.e., water-soluble, exchangeable, carbonate-bound and extractable at pH = 5, Fe-Mn oxide-bound, organic, sulfide-bound and residual. The sequential extraction steps proposed by Tessier have been widely used for metal partitioning in soil and sediments. The second procedure [21] divides metals into seven fractions, distinguishing between iron oxides and hydroxides and primary and secondary sulfides. This method has been adapted to the mineralogy of mine tailings from Cu-sulfide ores. A brief description of both procedures for 1 g of sample is given below.

The total mass of tailing was divided into six slices of equal thickness (0.013 m). The slice to be analyzed was homogenized before the analysis by sequential extraction.

#### 2.2.1. Sequential Extraction Method—Six Fractions

Soluble fraction: The sample was centrifuged for 2 h with 15 mL of deionized water at room temperature.

Exchangeable fraction: The residue of the soluble fraction was mixed with 8 mL of 1 mol/L MgCl_2_ at pH 7 and stirred for 1 h at room temperature.

Carbonate-bound fraction and/or acid-extracted fraction: The residue of the previous fraction was extracted with 8 mL of NaOAc (adjusted to pH = 5.0 with HOAc) by stirring for 5 h at room temperature.

Iron and manganese oxide-bound fraction: The residue of the carbonate-bound fraction was extracted at 96 °C with 15 mL of 0.04 mol/L NH_2_OH·HCl in 25% v/v HOAc for 6 h at room temperature with occasional stirring.

Organic matter and sulfide fraction: The residue of the previous fraction was extracted at 85 °C for 3 h with occasional stirring with a mixture of 3 mL 0.02 mol/L HNO_3_ and 5 mL 30% H_2_O_2_ adjusted to pH = 2.0 with HNO_3_. A second 3 mL aliquot of 30% H_2_O_2_ (pH = 2.0 with HNO_3_) was added, maintaining the same temperature and with occasional stirring. When the sample was cool, 5 mL of NH_4_OAc in 20% v/v HNO_3_ were added. The samples were diluted to a final volume of 20 mL and agitated continuously for 30 min.

Residual fraction: The residue of the previous fraction was digested with a solution of HCl-HNO_3_, with stirring for 16 h at room temperature [19,20].

#### 2.2.2. Sequential Extraction Method—Seven Fractions

Soluble fraction: The sample was centrifuged for 1 h with 50 mL of deionized water at room temperature.

Exchangeable fraction: The residue of the previous fraction was extracted by stirring with 1 mol/L NH_4_Ac adjusted to pH = 4.5 for 2 h at room temperature.

Fe(III) hydroxide fraction: The residue of the previous fraction was extracted with 0.2 mol/L ammonium oxalate at pH = 3.0 and stirred for 1 h at room temperature in the dark.

Fe(III) oxides fraction: The residue of the Fe(III) hydroxide fraction was extracted with 0.2 mol/L of ammonium oxalate adjusted to pH = 3.0 and placed in a 80 °C hot bath for 2 h.

Organic matter and secondary sulfides fraction: The residue of the previous fraction was mixed with 30% H_2_O_2_ and stirred for 1 h at 80 °C.

Primary sulfide fraction: The residue of the previous fraction was extracted with a boiling combination of KClO_3_ and HCl for 45 min, then boiling 4 mol/L HNO_3_ was added and the mixture was stirred for 40 min.

Residual fraction: The residue of the primary sulfide fraction was extracted by stirring with a mixture of HNO_3_ and HCl for 16 h at room temperature [19].

In both cases, after each extraction, the solid/liquid separation was carried out by centrifugation for 30 min. The supernatants were removed with a pipette and the sample was filtered with 0.8 μm polycarbonate membrane, diluted to 25 mL and analyzed for metal content. The residue was washed for 30 min before the next extraction.

Copper concentration in liquid samples was measured by atomic absorption spectrometry using a SpectrAA Varian spectrometer.

In the case of the tailing sample, no organic matter or carbonate fraction were expected. Therefore, these terms were omitted in the following text.

The reagents used in both methods were analytical grade, Merck, Kenilworth, NJ, USA: 30% hydrogen peroxide (ISO); magnesium chloride hexahydrate (Ph Eur, ISO); hydroxylamine hydrochloride (ACS, 98%); anhydrous sodium acetate (ACS, Ph Eur); di-ammonium oxalate monohydrate (Ph Eur, ACS, ISO); and acetic acid (96%).

### 2.3. Other Measurements

Copper concentration in solid samples was measured according to Danish Standard (1982) Determination of metals in water, sludge and sediments—general guidelines for determination by atomic absorption spectrometry in flame. Ds 259, as follows: 1 g of sample with 10 mL of distilled water and 10 mL of concentrated HNO_3_ was diluted to the mark in a 100 mL flask and autoclaved at 120 °C and 200 kPa for 1 h. The sample was cooled and filtered using filter paper with a pore size of 0.45 μm. The solution was analyzed using a SpectrAA Varian spectrometer.

pH was measured according to U.S. Environmental Protection Agency (US EPA), Method 9045. Water content was measured by weight loss after drying for 24 h at 105 °C in a furnace.

All measurements were carried out in three replicate samples and their average values are presented here.

## 3. Results and Discussion

Tailing samples (pH = 3.9) with an initial concentration of 1109 ± 32 mg Cu/kg of dry tailing were used in this research.

The copper concentrations associated with each fraction in both methods of sequential extraction for non-treated tailings are presented in Table 1 and Table 2.

As can be seen from Table 1 and Table 2, irrespective of the sequential extraction procedure used, close to 40% of copper was associated with the soluble fraction, copper total sulfides (primary and secondary) corresponded to 30% of the total copper and lower percentages corresponded to exchangeable and Fe oxide fractions.

The difference in the soluble fraction between both procedures could be attributed to the different times of extraction and water volumes.

The exchangeable fraction, which contains weakly adsorbed copper, is quite low compared to the total copper content as expected (close to 20% of the total copper). A higher fraction was extracted with ammonium acetate at pH = 4.5, compared to the use of a solution of magnesium chloride with pH = 7. This could be linked with the more acidic condition generated by ammonium acetate.

The extractable fraction at pH = 5 and Fe-Mn oxides in the case of the six-fraction procedure and Fe oxides and hydroxides in the case of the seven-fraction procedure cannot be directly compared due to the use of different extractant solutions, although it is possible to infer a higher concentration of copper compounds associated with iron hydroxides than oxides in the case of the second procedure.

The sulfide fraction in the case of the six-fraction procedure was 343.8 mg Cu/kg of dry tailing, which approximately corresponded to the sum of secondary and primary sulfide fractions in the case of the seven-fraction procedure, where the analysis indicated a concentration of 82.5 and 270.4 mg Cu/kg of dry tailing, respectively (total = 352.9 mg Cu/kg of dry tailing).

The residual fraction was determined by the same procedure. Copper concentrations differed by 1.3%.

The seven-fraction procedure gave additional information compared to the six-fraction procedure, especially when Fe oxides and hydroxide analysis was relevant. The two procedures showed similar results, with discrepancies that can be associated with the use of different extractants and times of extraction, thus the fraction pattern obtained by the six-fraction procedure was F1-1 > F1-5 > F1-6 > F1-2 > F1-3 > F1-4 and in the case of the seven-fraction procedure F2-1 > F2-6 > F2-7 > F2-2 > F2-5 > F2-3 > F2-4, showing the importance of the soluble fraction since, in the face of a more acidic pH, this part of copper will be liberated into the environment with the respective pollution problems.

The current efficiency for copper removal and the removal efficiency of the electrodialytic process are presented in Table 3.

The results of experiments showed that the studied process allows the removal of copper from pre-leached and electro-remediated tailings. As can be seen from Table 3, higher removal efficiencies for copper were obtained with a leaching solution with H_2_SO_4_ + HNO_3_. The variation between different pH levels was less than 5 percentage points, obtaining a 67.4% with pH = 1.9 and 62.1% with pH = 4.2. The lowest efficiency, close to 50%, was obtained with a leaching solution of ammonium chloride. Current efficiency behaves similarly to the copper removal efficiency, achieving 9% when a leaching solution of H_2_SO_4_ + HNO_3_ pH = 1.9 was used.

**Figure 1:**
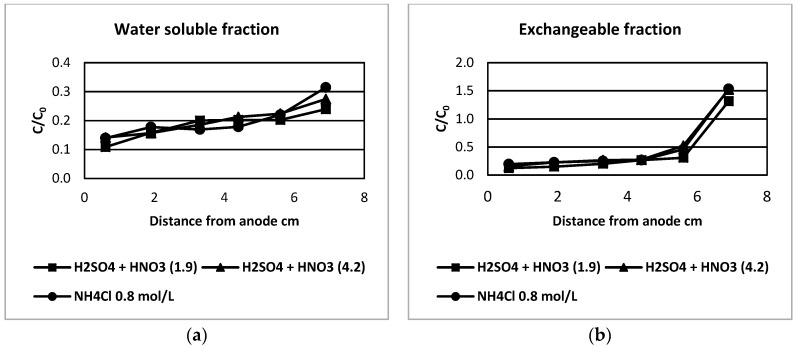

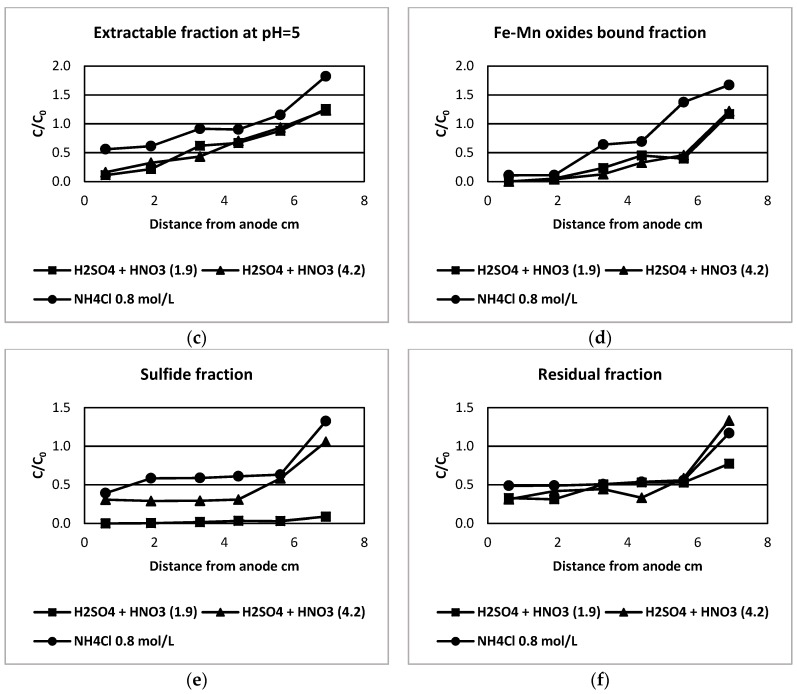
C/C_0_ ratio of copper concentration for slices closest to anode and closest to cathode for different pre-treatment solutions after electrodialytic remediation. Analysis of the six-fraction procedure, where the fractions are: (**a**) water soluble fraction, (**b**) exchangeable fraction, (**c**) extractable fraction at pH = 5, (**d**) Fe-Mn oxides bound fraction, (**e**) sulfide fraction and (**f**) residual fraction.

**Figure 2:**
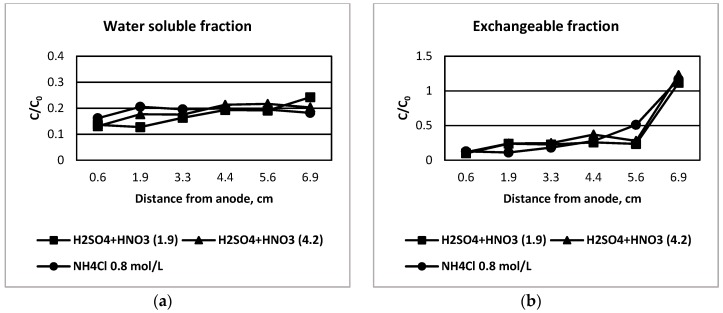

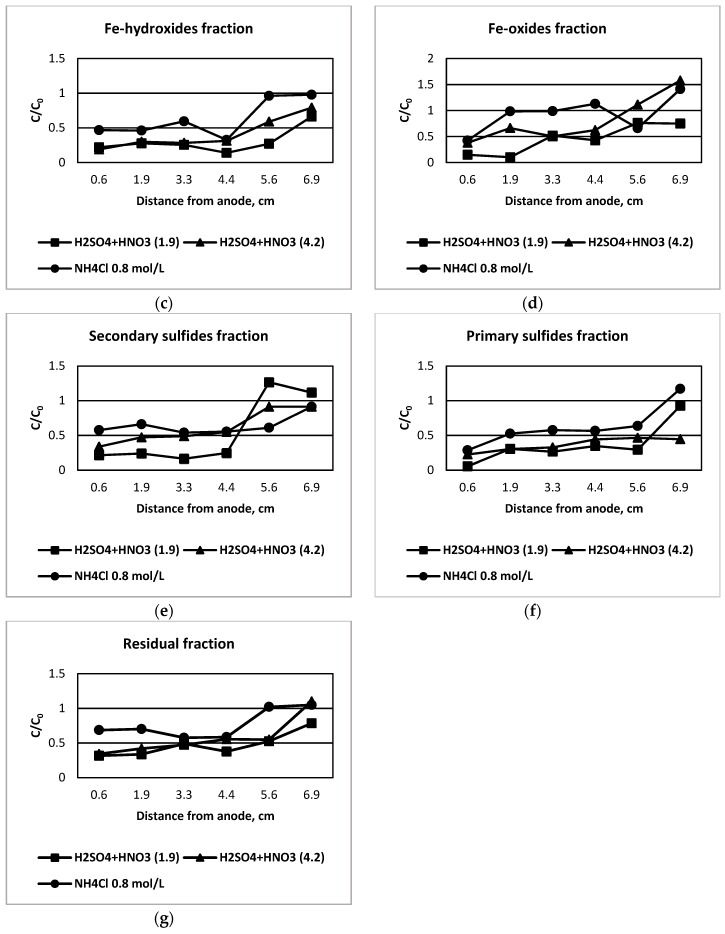
C/C_0_ ratio of copper concentration for slices closest to anode and closest to cathode for different pre-treatment solutions after electrodialytic remediation. Analysis of the seven-fraction procedure, where the fractions are: (**a**) water soluble fraction, (**b**) exchangeable fraction, (**c**) Fe-hydroxides fraction, (**d**) Fe-oxides fraction, (**e**) secondary sulfides fraction, (**f**) primary sulfides fraction and (**g**) residual fraction.

The ratio C/C_0_ is presented in Figure 1 and Figure 2, where C_0_ is the initial concentration of copper in the slice before all treatments and C is the concentration of copper in the same slice of tailing at the end of leaching and electrodialytic treatment for each analyzed fraction by the extraction sequential method, where the six-fraction procedure is presented in Figure 1 and the seven-fraction procedure in Figure 2.

The copper concentration associated with the soluble fraction, determined by both procedures, showed similar behavior. The analysis by the seven-fraction procedure gave lower concentrations of copper in all analyzed slices, which is consistent with the previously presented results. The fact could be linked to the total time of extraction by centrifugation. In any case, copper associated with this fraction is easily removed and it can be of concern in the case of the exposure of tailings to rain.

In the case of the exchangeable fraction, a similar trend was seen in both cases, although a different extractant solution and pH value was used. The general behavior was a higher removal in the complete cell except for the slice closest to the cathode, where copper accumulation was observed clearly, showing the movement of copper cations from anode to cathode.

The sulfide concentration determined by the sequential extraction of the six-fraction procedure should be close to the sum of primary and secondary sulfide corresponding to the seven-fraction procedure. In this respect, the total sulfide concentration was quite similar by both methods, as is presented in Table 1, and is equivalent to the part of the sample that is released in case of a more oxidative environment. The tendency of the concentration values between anode and cathode was consistent with a higher removal of copper closest to the anode and a slight accumulation towards the cathode for both procedures. A higher removal of copper associated with these fractions was obtained with the pre-treating solution corresponding to H_2_SO_4_ + HNO_3_ with pH = 1.9, followed by the same solution with pH = 4.2. Finally, with NH_4_Cl 0.8 mol/L the lowest removal was obtained.

The concentration of copper associated with the residual fraction showed similar results by both methods. The most notorious difference was with respect to the solution of NH_4_Cl 0.8 mol/L. Both methods indicated the best performance with H_2_SO_4_ + HNO_3_ solutions. The release of copper associated with this fraction was more difficult, although an increase in the removal was observed towards the anode.

The removal efficiencies of copper for each fraction and treatment solution after electrodialytic treatment are presented in Table 4 in the case of the six-fraction procedure and in Table 5 in the case of the seven-fraction procedure.

An easier removal was achieved in the case of copper associated with the soluble fraction with efficiencies higher than 80%. The exchangeable fraction of copper and copper associated with compounds released in acidic environments were removed with efficiencies between 50% and 60%, where a more acidic leaching solution showed the best performance. Soluble and exchangeable fractions will be easily released under little environmental changes. For this reason, these fractions have a high polluting potential. The treatment proposed allows much of the compounds associated to these fractions to be removed from the tailings.

In the case of sulfides, both methods showed the same trend with a higher removal with a mixture of H_2_SO_4_ and HNO_3_, pH = 1.9. However, the seven-fraction procedure allowed us to differentiate between secondary and primary sulfides, where primary sulfide removal showed removal efficiencies higher than secondary sulfides, 37% to 64% and 36% to 46%, respectively, depending on the leaching solution. Possibly, the greater presence of primary sulfides than secondary sulfides promoted these results, but it must be confirmed by subsequent studies.

## 4. Conclusions

Copper distribution in mine tailings pre-treated with three different leaching solutions and electro-remediated by electrodialysis was analyzed by two different procedures of sequential extraction. One of them divided the sample into six fractions and the other into seven fractions, contradistinguishing primary and secondary sulfides, iron oxides and hydroxides.

The studied treatment allowed us to remove copper with copper removal efficiencies between 53% and 67%, depending on the leaching solution used as pre-treatment. The best performance was obtained with the solution of H_2_SO_4_ + HNO_3_ adjusted to pH = 1.9 with a current efficiency close to 10%.

The results obtained from the application of both procedures of sequential extraction were consistent. Both procedures showed the same trend of copper concentration between anode and cathode, higher removal in the slices closest to the anode and lower removal closest to the cathode, even having an accumulation of copper in some cases.

Both methods indicated that most copper was associated with the soluble fraction, achieving for this fraction removal efficiencies close to 80% after the treatment.

The seven-fraction procedure gave more specific information, however, the higher removal of copper associated with primary sulfides than secondary sulfides needs to be more deeply studied.

The mobility and bioavailability of copper depends on the mineralogical and chemical form in which it occurs. The use of leaching or process solutions could also modify the mobility of copper. For these reasons, it is not possible to ensure the consistency of both procedures for all types of tailings. However, the usefulness of both procedures was demonstrated in the case of the studied tailing and it is possible to use them to predict the possible changes in the residue under specific conditions.

## Figures and Tables

**Table 1:** Copper concentration in mg Cu/kg of dry tailing in each fraction. Sample analyzed by the six-fraction procedure.

Fraction	Soluble F1-1	Exchangeable F1-2	Extractable at pH = 5.0 F1-3	Fe-Mn Oxides F1-4	Sulfides F1-5	Residual F1-6
mg Cu/kg of dry tailing	449.1 ± 14.1	77.6 ± 3.3	33.3 ± 1.5	16.6 ± 0.8	343.8 ± 13.1	188.5 ± 8.5

**Table 2:** Copper concentration in mg Cu/kg dry tailing in each fraction. Sample analyzed by the seven-fraction procedure.

Fraction	Soluble F2-1	Exchangeable F2-2	Fe Hydroxides F2-3	Fe Oxides F2-4	Secondary Sulfides F2-5	Primary Sulfides F2-6	Residual F2-7
mg Cu/kg of dry tailing	409.2 ± 19.1	90.3 ± 3.8	45.1 ± 1.8	25.5 ± 1.1	82.5 ± 3.7	270.4 ± 11.0	191.0 ± 9.1

**Table 3:** Copper removal efficiency and current efficiency after electrodialytic treatment according to leaching solution.

Leaching Solution	H_2_SO_4_ +HNO_3_ pH = 1.9	H_2_SO_4_ + HNO_3_ pH = 4.2	NH_4_Cl 0.8 mol/L pH = 5.5
Copper removal efficiency %	67.4	62.1	52.8
Current efficiency %	9.4	5.8	4.7

**Table 4:** Copper removal efficiency for each fraction. Sample analyzed by the six-fraction procedure.

Fraction	Soluble	Exchangeable	Extractable to pH = 5	Fe-Mn Oxides	Sulfides	Residual
H_2_SO_4_ + HNO_3_ pH 1.9	81.5%	60.6%	61.6%	37.6%	61.9%	50.5%
H_2_SO_4_ + HNO_3_ pH 4.2	80.1%	50.6%	63.9%	36.9%	52.8%	43.0%
NH_4_Cl 0.8 mol/L	80.0%	51.2%	23.4%	0.60%	31.2%	37.6%

**Table 5:** Copper removal efficiency for each fraction. Sample analyzed by the seven-fraction procedure.

Fraction	Soluble	Exchangeable	Fe Hydroxides	Fe Oxides	Secondary Sulfides	Primary Sulfides	Residual
H_2_SO_4_ + HNO_3_ pH 1.9	82.4%	63.7%	69.6%	54.9%	45.9%	63.5%	52.8%
H_2_SO_4_ + HNO_3_ pH 4.2	81.4%	58.6%	58.9%	19.0%	38.8%	63.3%	42.6%
NH_4_Cl 0.8 mol/L	81.0%	60.3%	36.8%	6.7%	35.8%	37.4%	22.9%

## References

[1] Alcalde J., Kelm U., Vergara D. (2018). Historical assessment of metal recovery potential from old mine tailings: A sutudy case for porphyry copper tailings Chile. Miner. Eng..

[2] Blessen S.T., Damare A., Gupta R.C. (2013). Strength and durability characteristics of copper tailing concrete. Constr. Build. Mater..

[3] Edraki M., Baumngartl T., Manlapig E., Bradshaw D., Franks D.M. (2014). Designing mine tailings for better environmental, social and economic outcomes: A review of alternative approaches. J. Clean. Prod..

[4] Ottosen L.M., Cristensen I.V., Pedersen A.J., Villumsen A., Lichtfouse E., Schwarzbauer J., Robert D. (2005). Electrodialytic Remediation of Heavy Metal Polluted Soil. Environmental Chemistry.

[5] Hansen H.K., Rojo A., Ottosen L.M. (2012). Electrodialytic remediation of copper mine tailings. Procedia Eng..

[6] Hansen H.K., Rojo A., Gutiérrez C., Jensen P.E., Ottosen L.M., Ribeiro A., Mateus E., Couto N. (2016). Electrokinetic remediation of copper mine tailings: Evaluating different alternatives for electric field. Electrokinetics across Disciplines and Continents: New Strategies for Sustainable Development.

[7] Hansen H.K., Lamas V., Gutiérrez C., Núñez P., Rojo A., Cameselle C., Ottosen L.M. (2013). Electro-remediation of copper mine tailings. Comparing copper removal efficiencies for two tailings of different age. Miner. Eng..

[8] Hansen H.K., Ribeiro A.B., Mateus E.P., Ottosen L.M. (2007). Diagnostic analysis of electrodialysis in mine tailing materials. Electrochim. Acta.

[9] Anju M., Banerjee D.K. (2010). Comparison of two sequential extraction procedures for heavy metal partitioning in mine tailings. Chemosphere.

[10] Ekmekyapar A., Oya R., Künkül A. (2003). Dissolution kinetics of an oxidized copper ore in ammonium chloride solution. Chem. Biochem. Eng..

[11] Liu W., Tang M., Tang C., He J., Yang S., Yang J. (2010). Dissolution kinetics of low grade complex copper ore in ammonia-ammonium chloride solution. Trans. Nonferr. Met. Soc. China.

[12] Okoro H., Fatoki O., Adekola F., Ximba B., Snyman R. (2012). A review of sequential extraction procedures for heavy metals speciation in soil and sediments. Sci. Rep..

[13] Yassir B., Fadeli Sana E., Mohy Edine K., Alain P. (2015). Study of potential environmental risk of trace metallic elements in mine tailings: Case of Draa Lasfar functional mine in Marrakech-Marocco. Afr. J. Agric. Res..

[14] Pourret O., Lange B., Bonhoure J., Colinet G., Decrée S., Mahy G., Séleck M., Shutcha M., Faucon M.P. (2015). Assessment of soil metal distribution and environmental impact of mining in Katanga (Democratic Republic of Congo). Appl. Geochem..

[15] Chen L., Wu J., Lu J., Xia C., Urynowicz M.A., Huang Z., Gao L., Ma M. (2018). Speciation, Fate and Transport, and Ecological Risks of Cu, Pb, and Zn in Tailings from Huogeqi Copper Mine, Inner Mongolia, China. J. Chem..

[16] Ribeiro A.B., Mexia J.T. (1997). A dynamic model for the electrokinetic removal of copper from a polluted soil. J. Hazard. Mater..

[17] Bacon J.R., Davidson C.M. (2008). Is there a future for sequential chemical extraction?. Analyst.

[18] Arenas-Lago D., Andrade M.L., Lago-Vila M., Rodríguez-Seijo A., Vega F.A. (2014). Sequential extraction of heavy metals in soils from a copper mine: Distribution in geochemical fractions. Geoderma.

[19] Lena Q.M., Gade N.R. (1997). Chemical fractionation of cadmium, copper, nickel and zinc in contaminated soils. J. Environ. Qual..

[20] Tessier A., Campbell P.G.C., Bisson M. (1979). Sequential extraction procedure for the speciation of particulate trace metals. Anal. Chem..

[21] Dold B. (2003). Speciation of the most soluble phases in a sequential extraction procedure adapted for geochemical studies of copper sulfide mine waste. J. Geochem. Explor..

[22] Xu Y.-H., Huang J.-H., Brandl H. (2017). An optimized sequential extraction scheme for the evaluation of vanadium mobility in soils. J. Environ. Sci..

[23] Javed M.B., Kachanoski G., Siddique T. (2013). A modified sequential extraction method for arsenic fractionation in sediments. Anal. Chim. Acta.

[24] Lazo A., Hansen H.K., Lazo P., Gutiérrez C. (2019). Application of a sequential extraction method for analyzing Cu distribution in pre-treated mine tailings after electrodialytic remediation. Int. J. Environ. Res. Public Health.

